# Age-related changes in Serum Growth Hormone, Insulin-like Growth Factor-1 and Somatostatin in System Lupus Erythematosus

**DOI:** 10.1186/1471-2474-5-37

**Published:** 2004-10-20

**Authors:** Charles W Denko, Charles J Malemud

**Affiliations:** 1 Department of Medicine/Division of Rheumatic Diseases, Case Western Reserve University School of Medicine, Cleveland, OH 44106-5076 USA; 2 Department of Medicine/Division of Rheumatic Diseases, and Department of Anatomy, Case Western Reserve University School of Medicine, Cleveland, Ohio, 44106-5076 USA

## Abstract

**Background:**

Systemic lupus erythematosus is an age- and gender-associated autoimmune disorder. Previous studies suggested that defects in the hypothalamic/pituitary axis contributed to systemic lupus erythematosus disease progression which could also involve growth hormone, insulin-like growth factor-1 and somatostatin function. This study was designed to compare basal serum growth hormone, insulin-like growth factor-1 and somatostatin levels in female systemic lupus erythematosus patients to a group of normal female subjects.

**Methods:**

Basal serum growth hormone, insulin-like growth factor-1 and somatostatin levels were measured by standard radioimmunoassay.

**Results:**

Serum growth hormone levels failed to correlate with age (r^2 ^= 3.03) in the entire group of normal subjects (i.e. 20 – 80 years). In contrast, serum insulin-like growth factor-1 levels were inversely correlated with age (adjusted r^2 ^= 0.092). Of note, serum growth hormone was positively correlated with age (adjusted r^2 ^= 0.269) in the 20 – 46 year range which overlapped with the age range of patients in the systemic lupus erythematosus group. In that regard, serum growth hormone levels were not significantly higher compared to either the entire group of normal subjects (20 – 80 yrs) or to normal subjects age-matched to the systemic lupus erythematosus patients. Serum insulin-like growth factor-1 levels were significantly elevated (p < 0.001) in systemic lupus erythematosus patients, but only when compared to the entire group of normal subjects. Serum somatostatin levels differed from normal subjects only in older (i.e. >55 yrs) systemic lupus erythematosus patients.

**Conclusions:**

These results indicated that systemic lupus erythematosus was not characterized by a modulation of the growth hormone/insulin-like growth factor-1 paracrine axis when serum samples from systemic lupus erythematosus patients were compared to age- matched normal female subjects. These results in systemic lupus erythematosus differ from those previously reported in other musculoskeletal disorders such as rheumatoid arthritis, osteoarthritis, fibromyalgia, diffuse idiopathic skeletal hyperostosis and hypermobility syndrome where significantly higher serum growth hormone levels were found. Somatostatin levels in elderly systemic lupus erythematosus patients may provide a clinical marker of disease activity in these patients.

## Background

Systemic lupus erythematosus (SLE) is the protean autoimmune disorder with strong familial penetrance. Immunologically, SLE is characterized by aberrations in T cell and B cell function [1,2], over-production of autoantibodies directed principally against nuclear antigens [3] as well as other tissue antigens, and deficiences in the complement system [4]. SLE is predominantly a disease of young females with peak incidence occuring between 20 and 40 yrs with a female to male ratio of 6–10:1 [5]. Although many of the principal pathophysiological changes associated with SLE indicate organ involvement consistent with vascular inflammation and immune complex deposition [6], several prominent SLE-related pathologic findings suggest systemic disturbances consistent with metabolic abnormalities [7]. However, surrogate blood or serum markers of systemic dysfunction such as erythyrocyte sedimentation rate and C-reactive protein levels, although frequently elevated in SLE compared to normal subjects are often uninformative and unreliable as surrogate markers of SLE disease activity [7].

We have shown that diverse rheumatic and musculoskeletal disorders, including osteoarthritis (OA) [8-12], diffuse idiopathic skeletal hyperostosis (DISH) [12,13] and hypermobility syndrome [14] as well as fibromyalgia [15] were characterized, in part, by elevated serum growth hormone levels. Growth hormone was also found sequestered in erythrocytes in OA and DISH patients at levels that significantly exceeded serum growth hormone levels [16] suggesting a putative mechanism by which "toxic" levels of growth hormone could be confined, or in cases of vascular inflammation, transported to joint synovial fluid or peripheral end-organs [11,16]. Further, medical therapy of OA and DISH principally with non-steroidal anti-inflammatory drugs (NSAIDs) which resulted in pain suppression and reduced stiffness as well as improved range of motion correlated with lower serum growth hormone levels consistent with levels found in normal subjects [10,13]. More recently, we showed that symptomatic rheumatoid arthritis (RA) patients were also characterized by elevated serum growth hormone levels [17], but treatment of RA with prednisone failed to significantly lower serum growth hormone levels.

Insulin-like growth factor-1 (IGF-1) synthesis is coupled to growth hormone via its capacity to stimulate hepatocyte IGF-1 production [11]. In several rheumatic and musculoskeletal disorders, elevated serum growth hormone was correlated with elevated IGF-1 levels [9,11,13,14] with OA [8-10,12] and RA [17] being notable exceptions. In the case of OA, IGF-1 levels are significantly lower compared to normal control subjects [8-10,12]. However, medical therapy of OA principally with NSAIDs resulted in growth hormone and IGF-1 levels approaching normal [10] whereas in DISH patients treated with NSAIDs, reduced serum growth hormone levels failed to result in concomitant changes in IGF-1 [13].

Somatostatin is a 14 amino acid polypeptide whose principal function is to regulate growth hormone release from the pituitary [18]. Elevated serum and synovial fluid somatostatin levels have been associated with inflammatory responses [19] most notably in RA [20]. A recent study showed that patients with symptomatic RA were, in part, characterized by a skewed upward serum growth hormone to somatostatin ratio [17].

The present study was performed to determine the extent to which serum growth hormone, IGF-1 and somatostatin levels were modulated in patients with SLE. A linear regression analysis was performed to determine the relationship between age and serum growth hormone and IGF-1 levels in a group of normal female subjects so that these values could be employed for comparison to a group of predominantly young, female SLE patients.

## Methods

All studies were performed at University Hospitals of Cleveland (UHC) and the Wade Park Veterans Administration Medical Center (VAMC), Cleveland, Ohio. The UHC and VAMC Institutional Review Boards approved the study design with the research protocol, which included informed consent, being in keeping with the Declaration of Helsinki. Normal subjects and SLE patients were all volunteers. SLE Patients met the clinical and laboratory criteria for the diagnosis of SLE according to previously published classifications [21]. Patients with co-morbid conditions such as diabetes mellitus or hyperglycemia were excluded from the normal subject group as was any normal individual with evidence for rheumatic disorders in family members. This information was obtained by questioning potential normal subjects.

Blood drawn by venipuncture was clotted at room temperature, centrifuged, serum aliquots separated and stored at -70°C until assayed. Blood samples were generally collected during an identical 3–4 hr morning period to normalize the potential contribution of growth hormone pulses and serum glucose levels to serum growth hormone determinations [8-10]. Serum samples were included for serum growth hormone, IGF-1 or somatostatin determinations only if glucose levels measured by the highly sensitive hexokinase assay [8-10] were between 65 and 135 mg/dl attained either by overnight fasting or a fast of at least 4 hours or more. Insulin levels were measured as previously described [8-10]. An insulin level in the range of 5–27 μU/ml was considered normal.

Basal serum growth hormone and IGF-1 levels were determined by standard radioimmunoassay (RIA) (INCSTAR, Stillwater, MN) as previously described [8-10]. The lower limit of detection for serum growth hormone by the RIA was 0.4 ng/ml [8-10]. Serum growth hormone levels in samples falling at or below the lower limit of detection were excluded from the statistical analysis. Basal somatostatin levels in serum of 112 normal subjects and 55 SLE patients stratified by age (i.e. <45, between 45 and 55 yrs and >55 yrs of age) were separately measured by RIA [17].

The 2-tailed T-test was employed to analyze the differences in means of serum growth hormone, IGF-1 and somatostatin concentrations in groups of unequal size where p < 0.05 was significant. The population sample size was sufficient to detect a 20% difference in serum growth hormone and IGF-1 levels between control subjects and SLE patients and a 15% difference in serum somatostatin levels. The relationship between serum growth hormone and IGF-1 levels as a function of age was analyzed from scatter plots by linear regression analysis employing SPSS 11.1 (SPSS, Inc., Chicago, IL) and SigmaPlot 8.0 (SPSS, Inc.) to calculate the adjusted r^2^-value and regression line, respectively.

## Results and discussion

Glucose and insulin concentration was determined in serum from normal female subjects and from patients with SLE. No normal female subjects were excluded from the study as a result of detecting hyperglycemia or hyperinsulinemia However, 2 SLE patients were excluded from the statistical analysis on this basis (data not shown).

Over the course of this study, SLE patients received medical therapy with NSAIDs, prednisone (10–60 mg/day), hydroxychloroquine sulfate or methotrexate as well as combinations of these drugs. No SLE patients were treated with azathioprine or cyclophosphamide during this study.

**Figure 1:**
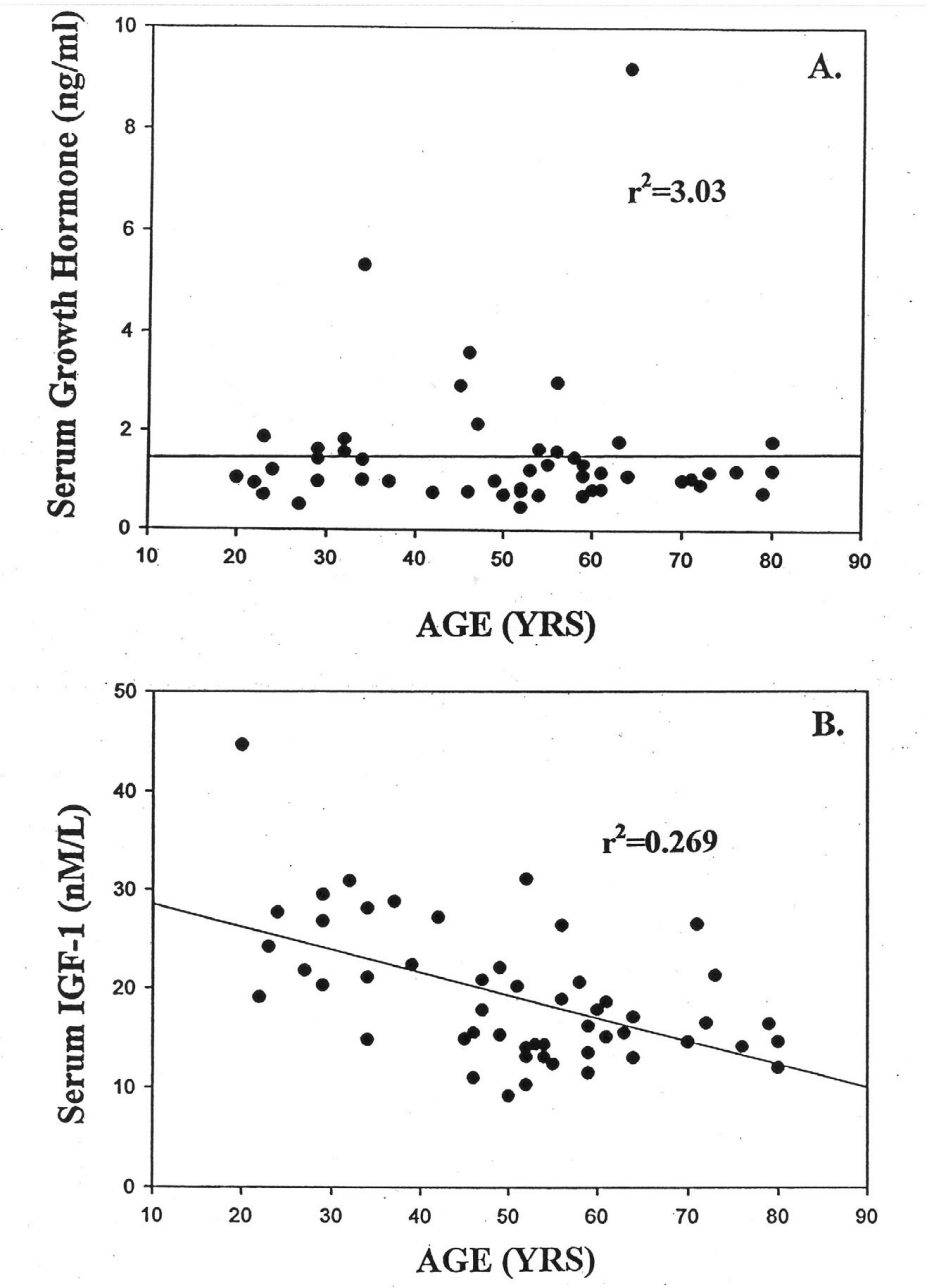
Linear regression analysis. Serum growth hormone levels (Panel A) or serum IGF-1 levels (Panel B) were plotted as a function of age in a normal female subject group. The results indicated that while serum growth hormone levels were not correlated with age (adjusted r^2 ^= 3.03), IGF-1 levels were inversely correlated to age (r^2 ^= 0.269).

Previously it was shown that basal serum growth hormone levels among normal male and female subjects did not significantly differ on the basis of age [16]. As noted, SLE has a high female to male prevalence ratio and is predominant in young females between 20 and 40 yrs of age [5,21]. Thus, it was critical to determine the extent to which serum growth hormone and IGF-1 differed among female normal subjects on the basis of age. Serum growth hormone levels did not correlate with age in normal female subjects between the ages of 20 and 80 (Figure 1A). However, a strong inverse correlation between age and IGF-1 levels (adjusted r^2 ^= 0.269) in this group of normal female subjects was found (Figure 1B).

**Figure 2:**
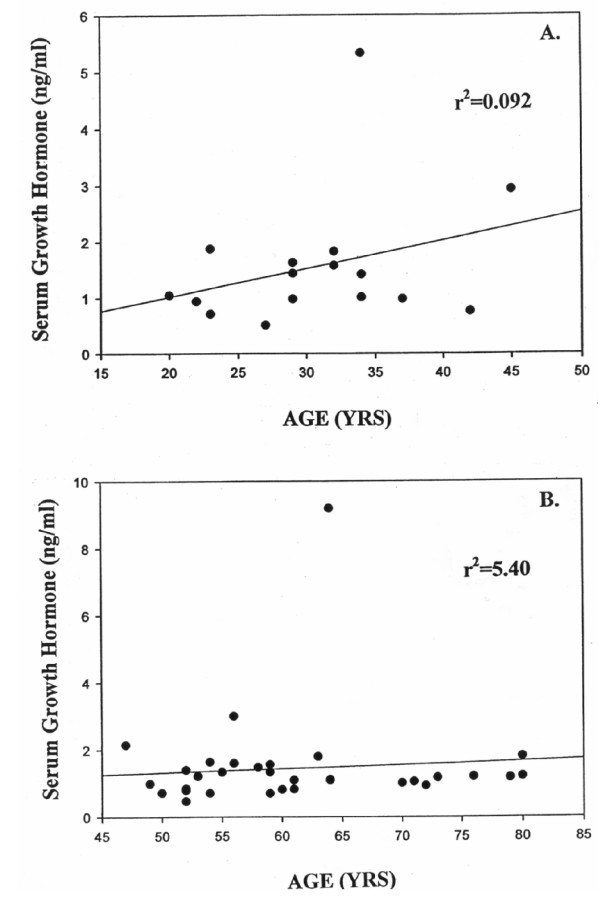
Linear regression analysis. Serum growth hormone levels were plotted as a function of age in young (age range, 20 to 46 yrs) normal female subjects (Panel A) or older (age range, 47 to 80 yrs) normal female subjects (Panel B). The results indicated that serum growth hormone levels correlated with age (r^2 ^= 0.092) only within the circumscribed group of younger normal female subjects.

In contrast to the results obtained from basal serum growth hormone measurements in the entire normal female subject population (Figure 1A), a strong direct correlation (adjusted r^2 ^= 0.092) between age (age, 30.8 ± 7.0, mean ± SD; 95% confidence, 3.74) and basal serum growth hormone levels in the young female normal subjects was found (Figure 2A). However, the correlation between age and basal serum growth hormone levels was weak in the older (age, 60.6 ± 9.4; mean ± SD; 95% confidence, 3.29) normal female subjects (Figure 2B).

Based on the above considerations, basal serum growth hormone and IGF-1 concentration was determined in study groups subdivided by age in normal female subjects and these values compared with basal serum growth hormone and IGF-1 levels in SLE patients. The results showed that SLE was not characterized by elevated serum growth hormone whether or not all normal female subjects or age-matched normal female subjects were employed as the comparison group (Table 1). Serum IGF-1 levels were significantly lower in the normal female subject group compared to SLE patients (Table 1), but there was no significant difference if serum IGF-1 levels in the SLE group were compared to serum IGF-1 levels in the age-matched normal female group (Table 1).

A trend towards elevated somatostatin levels in normal subjects as a function age was previously found [17]. In the present study, there was also a trend towards elevated serum somatostatin levels in the <45 yr old SLE patient group or 45 – 55 yr old group compared to their age-matched normal counterparts (Table 1). However, a significant difference was found only in the older (>55 yrs) SLE patients compared to their age-matched control counterparts (Table 1).

The results of the present study emphasized the critical requirement to control for age and gender when basal serum growth hormone and IGF-1 levels in normal subjects are compared to patients with autoimmune musculoskeletal diseases which, like SLE, are characterized by a strong age and gender association.

Several studies from our laboratory have consistently shown basal serum growth hormone to be higher in females than in males [8,9,14]. One study, in particular, examined the correlation between age, gender and race with basal serum growth hormone and concluded that, in general, older Causcasian women had slightly higher growth hormone levels compared to older African-American women [8]. However, in that study (8) no statistical differences were shown when serum growth hormone levels in young Caucasian women (age, 28 ± 6; mean ± SD) were compared to serum growth hormone levels in African-American women (age, 34 ± 10). This finding is particularly noteworthy to studies of SLE because, in most cases, SLE onset is prominent among young females during their reproductive years, and African-American women are over-represented in the SLE patient population [21].

The present analysis also extends the results of previous studies [8,9,14] and partially supports the conclusions of Ghigo *et al*. [22] who showed that basal growth hormone levels were similar in young and older individuals. Ghigo *et al*. [22] further suggested that the somatotroph response in young versus older individuals to the combined administration of arginine and growth hormone-releasing substance also did not vary with age.

In contrast, the present results do not support the conclusions that growth hormone decreases as a function of age as reported by Kelijman [23]. In fact, the results of the present study showed a strong correlation between age and serum growth hormone only in the circumscribed young normal female (age 20 – 46 yrs) group (Figure 1A).

The decrease in basal serum IGF-1 levels with age (Figure 1B) confirmed previous studies by Hochberg *et al*. [24] who studied patients with osteoarthritis of the knee as well as earlier studies by Ghigo *et al*. [22] who reported a significant difference in IGF-1 levels between young and older individuals. Thus, it was not unexpected that basal serum IGF-1 levels in SLE was significantly elevated when compared to basal serum IGF-1 levels in the general population of normal subjects, but not so, when basal serum IGF-1 levels from SLE patients were compared to their age-matched counterparts (Table 1). In this regard, Bennett *et al*. [25] also failed to find differences in serum IGF-1 levels when normal subjects (age, 45.1 ± 8.6) were compared to 15 age-matched SLE patients (age, 42.5 ± 7.0).

The relationship between putative abnormalities in the hypothalamic-pituitary axis, systemic disturbances and SLE pathogenesis and progression remains conjectural. In this regard, Rovensky *et al*. [26] found no correlation between plasma prolactin, growth hormone, interleukin-6, cortisol or C-reactive protein in adult SLE patients. However, studies by Chikanza *et al*. [27] reached a different conclusion. They suggested that a "pro-inflammatory hormonal bias" existed in juvenile SLE which was identical to adult SLE. They also concluded that the role of the neuroendocrine-immune system in adult SLE was, at the present time, limited to deficiencies in prolactin. Of note, two recent case reports suggested a link between growth hormone and exacerbation of lupus nephritis in a male teenager with SLE [28] as well as in juvenile SLE [29] where when growth retardation treated with growth hormone was terminated, clinical improvement in lupus symptoms was observed. These findings suggested that exogenously-administered growth hormone may result in "toxic" levels of growth hormone accompanied by lupus "flares" with progressive autoimmune dysfunction.

A recent study from this laboratory showed that the growth hormone to somatostatin ratio was skewed upward in patients with RA [17]. In the present study, somatostatin levels in the age groups encompassing the average age of the SLE patients were not different from than of normal subjects (Table 1). Although previous studies have suggested that somatostatinergic activity increased with age [20], the present analysis (Table 1) does not support that view (at least from measurements of basal somatostatin levels) as lower somatostatin levels in the older SLE patients reached statistical significance when compared to age-matched controls with the caveat that the present study did not relate changes in somatostatin to SLE disease activity.

Although somatostatin may alter growth hormone effects and immune responses in chronic autoimmune diseases, the relationship between somatostatin and "specific" somatostatin receptor (sSR) in SLE remains to be elucidated. In this regard, van Hagen [30] showed that 97% of patients with sarcoidosis, 100% of patients with tuberculosis or Wegener's granulomatosis, 75% of patients with Sjogren's syndrome but only 50% of SLE patients exhibited sSRs on mitogen-activated human peripheral lymphocytes compared to 97% in normal individuals. Of note, somatostatin receptor levels appeared to be unrelated to disease progression or remission. In the present study, a trend towards reduced serum somatostatin levels was seen only in the older SLE patients (Table 1). As functional somatostatin may change in autoimmunity and result in altered growth hormone release, reduced somatostatin levels could also influence basal levels of growth hormone in elderly SLE patients. Thus, changes in somatostatin could be one of several environmental stress factors resulting in the progression of clinically active disease in older SLE patients [31].

The therapeutic implications and diagnostic utility of serum growth hormone, IGF-1 and somatostatin measurements in SLE as well as in other musculoskeletal disorders appears central to assigning a role for these factors in disease progression. Serum growth hormone remained elevated in some DISH and OA patients where clinical symptoms were significant [9,11,12]. Thus, single serum growth hormone determinations appear to accurately reflect a pattern of serum growth hormone levels associated with these clinical disorders. Further, improvement in the clinical symptoms in OA and DISH patients with medical therapy [10,13] resulted in a sustained reduction in serum growth hormone levels reaching levels comparable to those found in normal subjects. Although longitudinal measurements of serum somatostatin in SLE and other rheumatic diseases have not yet been performed, reduced somatostatin levels appear to be most strongly associated with joint inflammation (as was seen in RA) [17] as well as in older patients (>55 yrs) with the inflammatory complications of knee OA (Denko and Malemud, submitted). Thus, it could be informative if elevated somatostatin levels correlated with clinical improvement in SLE patients.

## Competing interests

The authors declare that they have no competing interests.

## Authors' contributions

CWD participated in the design of the study and performed the clinical analysis. CJM participated in the design of the study and performed the statistical analysis. All authors read and approved the final manuscript.

## Pre-publication history

The pre-publication history for this paper can be accessed here:



## Figures and Tables

**Table 1:** Serum Growth Hormone (GH), Insulin-like Growth Factor-1 (IGF-1) and Somatostatin (SOM) levels in normal female subjects and Systemic Lupus Erythematosus (SLE) patients

Group	Age (yrs)*	GH ng/ml)*	IGF-1 (nM/L)*	SOM (pg/ml)*	p-value
All Normals (35)	57.1 ± 13.1	1.17 ± 0.40	14.9 ± 3.6		
(77)	<45			25.2 ± 11	
(17)	45–55			32.6 ± 12	
(18)	>55			36.2 ± 10	
Age- Matched Normals (18)	30.8 ± 7.1**	1.58 ± 1.16**	25.0 ± 7.3^†^		
SLE (17)	35.9 ± 8.6^†^	1.45 ± 0.88^†^	22.8 ± 7.1**		
(22)	< 45			29.8 ± 12	P > 0.05
(12)	45–55			35.4 ± 14	P > 0.05
(21)	>55			29.9 ± 9	P < 0.05
AMNv.SLE	**P > 0.05	**P > 0.05	**P > 0.05		
ANv.SLE	^†^P < 0.001	^†^P > 0.05	^†^P < 0.001		

• Mean ± SD

• N: number of samples

AMN: age-matched normal subjects

AN: all normal subjects

SLE: systemic lupus erythematosus patients
